# A Systematic Review of Herbal Medicine for Chemotherapy Induced Peripheral Neuropathy 

**DOI:** 10.1155/2018/6194184

**Published:** 2018-02-14

**Authors:** Hyeonseok Noh, Seong Woo Yoon, Bongki Park

**Affiliations:** ^1^Department of Clinical Korean Medicine, Graduate School, Kyung Hee University, Seoul, Republic of Korea; ^2^Department of Korean Internal Medicine, Korean Medicine Cancer Center, Kyung Hee University Hospital at Gangdong, College of Korean Medicine, Kyung Hee University, Seoul, Republic of Korea; ^3^Department of Korean Internal Medicine, Ilsan Oriental Hospital, Dongguk University, Gyeonggi, Republic of Korea

## Abstract

**Background:**

Chemotherapy-induced peripheral neuropathy (CIPN) is a common adverse effect in cancer patients. The aim of this review was to assess the effectiveness of herbal medicine in preventing and treating CIPN.

**Methods:**

Randomised controlled trials were included in this review. Extracting and assessing the data independently, two authors searched 13 databases.

**Results:**

Twenty-eight trials involving 2174 patients met the inclusion criteria. Although there were some exceptions, the methodological quality was typically low. Seventeen trials reported the incidence rate of CIPN assessed by various tools and 14 showed a significant difference regarding the decrease of the incidence rate between the two groups. For clinical improvement, 12 trials reported it using various tools and 10 showed a significant difference between two groups. Two cases of adverse events occurred in one trial; the other nine trials reported no adverse events.

**Conclusions:**

We found that herbal medicines in combination with and/or without other therapies potentially have preventive or therapeutic effects on CIPN. However, conclusions cannot be drawn because of the generally low quality of the methodology, the clinical heterogeneity, and the small sample size for each single herbal medicine. Trials that are more rigorous and report sufficient methodological data are needed.

## 1. Introduction

Chemotherapy induced peripheral neuropathy (CIPN) is a common and stubborn adverse effect of chemotherapy agents such as platinum agents, taxanes, vinca alkaloids, epothilones, thalidomide, and bortezomib [1, 2]. Approximately 20 to 40% of cancer patients suffer from CIPN [3, 4]. Although the mechanisms of CIPN are not fully understood, recent research has shown that the dorsal root ganglion (DGR) of the neural cell bodies is the most common target of chemotherapy agents [1, 5]. Microtubule disruption (in the case of taxanes, epothilones, vinca alkaloids, and bortezomib), the nerve terminal damage (in the case of vincristine and taxanes), and dysregulation of neutrophins (in the case of thalidomide and bortezomib) also contribute to inception of CIPN [4, 5]. Clinical features of CIPN vary according to the chemotherapy agents used and the site of their action. CIPN is primarily manifested in symptoms of sensory neuropathy, including pain, tingling, numbness, and temperature sensitivity in the hands and feet. Occasionally, when paclitaxel and vincristine are used, the motor neuron can be damaged [4–6], and autonomic nerves may be affected by the use of vinca alkaloids [5, 7]. Although CIPN is generally dose dependent, it can occur at any time during chemotherapy or after the end of treatment, and it can take different form. Oxaliplatin induced neurotoxicity can occur in a clinically acute and chronic form: the acute form is thought to occur as a result of a dysfunction of nodal axonal voltage, and chronic toxicity is thought to be associated with functional changes in the DGR [5, 8]. Late presentations are commonly associated with cisplatin [1, 9].

Because of these clinical features, CIPN decreases quality of life (QoL). It also enforces treatment delays and dose reductions of chemotherapeutic agents, thereby limiting their efficacy [1, 5]. Numerous pharmacological and nutraceuticals options have been tested for the treatment of CIPN including amifostine, glutamine, glutathione, oxcarbazepine, antiepileptics, venlafaxine, vitamin B6, vitamin E, omega-3 fatty acids, acetyl-L-carnitine, and duloxetine [1, 5, 6, 10, 11]. Although these agents have been shown to be effective in the prevention and treatment of CIPN, the data are insufficient to demonstrate the efficacy of pharmacological drugs and nutraceuticals [1, 5, 12].

Herbal medicine has been developed and used widely in East Asia; it is based on the unique theories of Yin and Yang, the five elements, and the visceral manifestation theory [13]. In modern times, herbal medicine is prescribed as therapeutic agents and as an adjunctive method for managing diverse diseases, including cancer [14, 15]. Numerous experimental and clinical studies have investigated the effectiveness of herbal medicine and have shown its potential effectiveness in managing CIPN through the action of antioxidants and the enhancement of nerve growth [16, 17]. Although some systematic reviews have been published on the effectiveness of herbal medicine for oxaliplatin induced peripheral neuropathy [18, 19], to the best of our knowledge, there is no systematic review of randomised controlled trials (RCTs) for the use of herbal medicines in CIPN, regardless of the chemotherapeutic agents or the herbal medicine. To assess the effectiveness of herbal medicines for CIPN, we therefore conducted this systematic review of RCTs without seeking to exclude any particular chemotherapeutic agents or herbal medicines.

## 2. Methods

### 2.1. Search Strategy

We searched the following 13 electronic databases, with no limitations on the publication date or the language: MEDLINE, the Cochrane Central Register of Controlled Trials (CENTRAL), EMBASE, AMED, the China National Knowledge Infrastructure (CNKI), the Wanfang Database, the CQVIP Database, and six Korean medical databases (Korean Studies Information, DBPIA, the Korea Institute of Science Technology Information, the Research Information Centre for Health Database, the Korean Traditional Knowledge Portal, and KoreaMed).

The search strategy was developed for MEDLINE (see Appendix A) and CNKI (see Appendix B). We also modified it for each database, based on the search terms. We completed the searches through 17 May 2017.

We registered the protocol of this review in PROSPERO (Available at https://www.crd.york.ac.uk/PROSPERO/display_record.asp?ID=CRD42017054369).

### 2.2. Eligibility Criteria

We included RCTs that investigated herbal medicine for preventing and treating CIPN in cancer patients, regardless of time or language. The participants included in the study met the following criteria: (1) age 18 years or older, (2) patients diagnosed with cancer who had received chemotherapy, regardless of type of cancer, sex, race, or geographic location, and (3) the CIPN diagnosed by clinical assessment or additional investigation, such as nerve conduction velocity. We included data from parallel-group studies for the meta-analysis. In the case of crossover trials, only the first treatment period data was analysed. If we were not able to separate the results of the first and second periods in the crossover trial, we excluded the study. The RCTs in which the data in the methods or the results section were unreliable or unavailable were excluded from the studies.

We included all types of herbal medicines and their major ingredients used for medicinal purposes, regardless of their origin, the composition of prescription, the mode of delivery (e.g., oral, intravenous infusion using fluid, intravenous injection, fumigation, external application, or hand and foot baths), and the dosage or the duration of the treatment. The comparators included no treatment, a placebo, and conventional therapeutic agents. We excluded those trials that used the other type of herbal medicine as a comparison and those that did not use the same baseline therapy.

The primary outcome measures were clinical improvement, incidence rate, and nerve conduction study (NCS). The clinical improvement and the incidence rate were measured by objective methods, including such things as the National Cancer Institute Common Terminology Criteria for Adverse Events (NCI-CTCAE) grade and Levi's grade. We assessed the clinical improvement using three grades with reference to Deng et al.'s 2016 article [19]: complete remission (CR), partial remission (PR), and being not perceptible (NP). CR is completely diminished symptoms and/or the CIPN grade has been reduced to zero. PR is the improvement of symptoms and/or a reduction of more than one grade. NP is either no reduction of the grade or no change of the symptoms, in comparison to the case before treatment. The effective rate is the sum of CR and PR. The NCS included motor and sensory nerve conduction velocity assessed by validated methods. The secondary outcome measures involved the patients' quality of life (QoL), as assessed by objective instruments and adverse effects. We also included information regarding adverse effects induced by chemotherapy.

### 2.3. Data Extraction and Assessment of Methodological Quality

The two authors (Noh and Park) assessed the titles and abstracts of the studies retrieved from electronic databases and each determined their eligibility for inclusion independently. Hard copies of the relevant studies were retrieved and then the authors used a standard data extraction form to extract the data independently. The form included methodology, participants, intervention, periods of treatment, outcomes, and conclusions. The two authors (Noh and Park) assessed the risk of bias using the Cochrane risk of bias tool [20] and the following items: random sequence generation (selection bias), allocation concealment (selection bias), blinding of participants and personnel (performance bias), blinding of outcome assessment (detection bias), incomplete outcome data (attrition bias), selective outcome reporting (reporting bias), and other biases. We evaluated the domains as “Yes,” “No,” or “Unclear,” according to the Cochrane risk of bias criteria. When there was a discrepancy among the reviewers, a consensus was reached through discussion.

### 2.4. Data Analysis and Synthesis

We used Review Manager 5.3 software to perform our data analyses. We used the risk ratios (RRs) with 95% confidence intervals (CIs) for dichotomous data and the mean difference (MD) with 95% CIs for continuous data. A meta-analysis was planned, which would be conducted using a random effect model, if there was sufficient data to pool. We planned to conduct a standardised mean difference analysis (SMD), with 95% Cis, in the case of the use of different measurement scales for continuous data. Heterogeneity was tested using *I*^2^. In the case of heterogeneity, we planned to analyse the subgroup analyses. Since the studies did not meet the qualifications, neither publication bias nor sensitivity analyses were performed.

## 3. Results

### 3.1. Description of Studies

**Figure 1:**
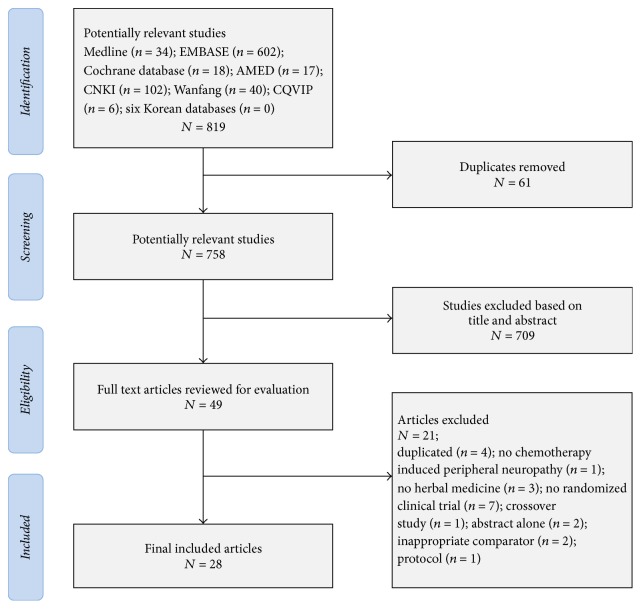
Flow diagram of study selection.

We identified 819 articles from the 13 electronic databases in the primary searches (Figure 1). After reading the titles and abstracts, we excluded 61 duplicate articles and we removed 758 that did not meet the inclusion criteria. We retrieved the full texts of 49 articles for further evaluation. Of the 49 articles, 21 were excluded, three were duplicates, one did not concern CIPN, three did not use herbal medicine as intervention, and six were not randomised. In the end, a total of 28 trials were included for this review [21–48]. Of those, 18 trials investigated herbal medicines during the period of chemotherapy [21–38] (Table 1) and 10 tested herbal medicine in patients with CIPN after chemotherapy [39–48] (Table 2).

### 3.2. Study Characteristics

A total of 2174 patients in 28 trials were included; 72 patients were dropped from the study. The mean of the participant sample size was 77.64 people (range: 31 to 186). Eighteen trials studied gastrointestinal cancer, six tested patients with various types of cancer, two studied ovarian cancer or endometrial cancer, and one trial each tested patients with breast cancer and multiple myeloma. The duration of the treatment ranged from 7 days to 40 weeks. All trials described baseline characteristics that were comparable between the intervention groups with the control groups. Although three trials tested herbal medicine during the period of chemotherapy, they did not describe the accurate treatment period [28, 34, 38].

Twenty-six different formulas were investigated in 28 trials (Table 3). The oral dosage form of herbal medicine, including decoction or granules, was used in 12 trials. In 11 trials, the herbal formula was used as some form of hand or foot bath. One trial applied herbal medicine through intravenous (IV) infusion and another applied it through fumigation. Three trials investigated oral herbal medicine combined with hand and foot baths of herbal medicine or the fumigation of herbal medicine. Goshajinkigan granules were used in five trials [24, 28, 30, 35, 43] and two used a guilong tongluo decoction [21, 32]. The other 24 formulas were investigated in 21 trials.

An oxaliplatin based regimen was used to treat cancer patients in 20 trials. A paclitaxel and carboplatin (PC) regimen was used in two trials [27, 43] and a docetaxel based regimen was used in one [28]. CIPN patients were treated by various types of chemotherapy regimen in four trials [40–42, 46]. One trial did not report on the chemotherapeutic agents in detail [48]. Regarding comparators, vitamin B12 was used in four trials [25, 28, 41, 46], glutathione was used in two [23, 26], a placebo was used in three [32, 35, 45], and simple hand and foot baths using warm water were used in three [36, 39, 40]. The other 16 trials compared interventions with no treatment. In seven trials, the common treatments were used in the treatment and control groups [22, 31, 42–44, 47, 48].

Regarding the outcome measurements, six assessment tools were used in assessing the incidence rate or clinical improvement: NCI-CTC grade (15 trials), Levi's grade (6 trials), Neurotoxicity Criteria of Debiopharm scale (DEB-NTC scale; 2 trials), World Health Organization grade (WHO grade; 2 trials), Functional Assessment of Cancer Therapy/Gynecological Oncology Group-Neurotoxicity (FACT/GOG-Ntx; 1 trial), and the Functional Assessment of Cancer Therapy-Taxane (FACT-Taxane; 1 trial). Four trials used the researcher's own criteria to assess the incidence rate or clinical improvement. Four trials reported on nerve conduction studies [33, 38, 42, 48] and one reported the current perception threshold (CPT) [43]. Nine trials reported on QoL [21, 23, 26, 33, 37, 40, 41, 43, 46]. For adverse events, 10 trials reported adverse events for interventions [30, 33, 36, 37, 39–41, 45–47] and eight reported on the adverse chemotherapy induced events when the participants were treated by chemotherapy combined with intervention treatments [21, 23, 24, 26, 28, 32, 35, 36].

### 3.3. Risk of Bias in the Included Studies

**Figure 2:**
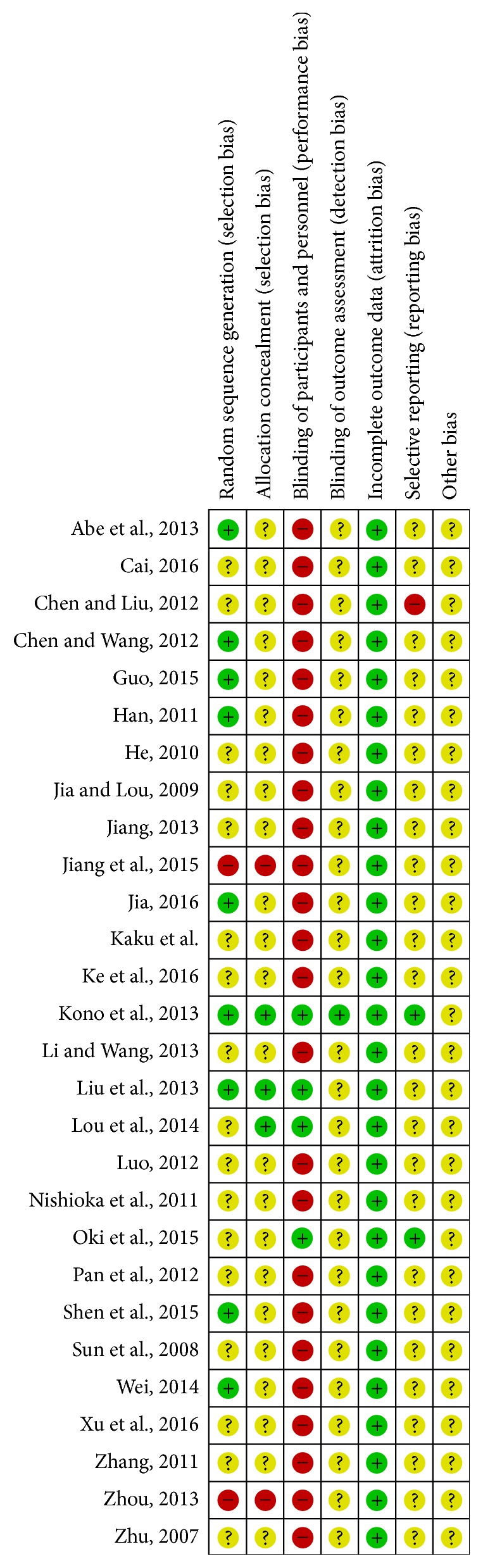
*Risk of bias summary: review of authors' assessment about each risk of bias item for each included study*. “+”: low risk of bias; “?”: unclear risk of bias; “−”: high risk of bias.

Nine trials reported the random sequence using a random number table or a computer number generator [25, 26, 28, 30, 33, 37, 40, 46, 47]. Two trials described an inadequate sequence generated by the date of admission [34, 44]. The other 17 trials did not report their random sequence. One trial described allocation concealment done in patients and investigators [30] and two used an opaque sealed envelope [26, 32]. Four trials reported double blinding of participants and investigators [30, 32, 35, 45]. Not all of the 28 trials described whether the outcome assessors were blinded or not, although one trial blinded the data collectors. Nine trials reported dropouts [23, 30, 33, 35, 39, 40, 43, 45, 46] and one applied an intention to treat analysis [30]. For the other six trials, we judged that the proportion of missing outcomes was not sufficient to have a clinically relevant impact on the intervention effect estimate. The remaining 21 trials did not miss outcome data. Two trials reported their prespecified protocols [30, 35]. Overall, with some exceptions, the methodological quality was not promising (Figure 2).

### 3.4. Effects of Interventions

We divided the 28 included trials into two categories: (1) herbal medicine investigated to prevent CIPN in cancer patients receiving chemotherapy (18 RCTs, Table 1) and (2) herbal medicine investigated as therapeutic agents in cancer patients with CIPN (10 RCTs, Table 2).

We analysed the first category as three subgroups, according to the dosage form of the herbal medicine: (1) herbal medicine for hand and foot baths or fumigation, (2) herbal medicine for intravenous infusion, and (3) herbal medicine for oral dosage form. The second category was analysed as two groups: (1) herbal medicine for hand and foot baths or fumigation and (2) herbal medicine for the oral dosage form. We did not pool the data due to clinical heterogeneity for the 21 trials that investigated the differences in herbal medicines. Since different comparators, different outcome measures, or different common treatments were used, we did not perform a meta-analysis for the five and two trials that investigated goshajinkigan granules and the guilong tongluo decoction, respectively.

### 3.5. Preventive Effects: Herbal Medicine for Hand and Foot Baths or Fumigation

Seven trials compared herbal medicine for hand and foot baths or fumigation with vitamin B12, hand and foot baths of warm water, and no treatment [25, 27, 29, 31, 33, 34, 36]. Six trials reported the incidence rate and all of the trials showed that herbal interventions were significantly effective in decreasing the incidence rate, as compared with the comparators: huoxue tongluo formula, Guo's specified formula, wenjing huoxue formula, siteng yixian formula, yiqi huayu formula for hand and foot baths combined with chaihu jia longgu muli decoction, and Li et al.'s specified formula for hand and foot baths combined with buyang huanwu decoction [25, 27, 31, 33, 34]. One trial referred to NCS and the fumigation of Guo et al.'s specified formula showed significant effectiveness in nerve conduction velocity and amplitude, as compared with warm water baths [33]. In addition, fumigation of Guo's specified formula showed significant improvement in the Karnofsky Performance Scale (MD 3.43, 95% CI 0.84 to 6.02) [33].

### 3.6. Preventive Effects: Herbal Medicine for IV Infusion

One trial investigated the preventive effects of Astragali Radix through IV infusion in comparison to no treatment and to glutathione [26]. Astragali Radix through IV infusion showed a significant reduction of the incidence rate and improvement of KPS (MD 7.68, 95% CI 4.29 to 11.07) in comparison to no treatment. However, in comparing Astragali Radix and glutathione, no significant differences were found in the incidence rate or the KPS.

### 3.7. Preventive Effects: Herbal Medicine for Oral Dosage Form

Ten trials tested the preventive effects of the six different herbal medicines in comparison to no treatment, vitamin B12, and placebo [21–24, 28, 30, 32, 35, 37, 38]. Ten trials reported the incidence rate of CIPN. For goshajinkigan granules, although Nishioka et al. reported significant effects on the reduction of the incidence rate [24, 28], Kono et al.'s results showed no significant deference between the intervention groups and the control groups [30, 35]. For the guilong tongluo decoction, Zhu et al. reported a significant reduction in the incidence rate in comparison to no treatment and a placebo [21, 32]. The yiqi huoxue decoction combined with neurotropin, yiqi wenjing tongluo decoction, shaoyao gouteng muer decoction, and tanshinone II showed a significant reduction in the incidence rate in comparison to the comparators [22, 37, 38]. Meanwhile, tanshinone II showed significant effects on motor nerve conduction velocity (MNCV) and sensory nerve conduction velocity (SNCV) in comparison to no treatment (MNCV; MD −8.30, 95% CI −10.72 to −5.88) (SNCV; MD −5.00, 95% CI −6.49 to −3.51) [38]. Three trials reported QoL using KPS and their results showed significant improvements between the intervention groups and the control groups [21, 23, 37].

### 3.8. Therapeutic Effects: Herbal Medicine for Hand and Foot Baths or Fumigation

Eight trials investigated the therapeutic effects of nine different herbal medicines for hand and foot baths or fumigation in comparison to warm water baths, vitamin B12, placebos, or no additional treatment [39, 40, 42, 44–48]. Eight trials reported clinical improvements of CIPN using NCI-CTC or the researcher's own criteria and all of the eight trials reported a significant difference between the two groups. The wenloutong formula (NCI-CTC grade; RR 2.90, 95% CI 1.68 to 5.02) [39] and the wenjing huoxue tongluo formula (NCI-CTC grade; RR 1.94, 95% CI 1.25 to 3.00) [40] were as significantly effective as warm water baths. The effectiveness rate of the wenjing tongluo formula was significantly improved in comparison to the hand and foot baths of placebo (NCI-CTC grade; RR 2.13, 95% CI 1.32 to 3.42) [45]. The effectiveness rate of the wenyang tongbi formula presented a significant improvement in comparison to vitamin B12 (NCI-CTC grade; RR 1.43, 95% CI 1.05 to 1.95) [46]. In addition, the modified huangqi guizhi wuwu formula combined with vitamin B12, aidi injections, and thymalfasin showed significant effects in improving the effectiveness rate in comparison with vitamin B12, aidi injections, and thymalfasin (NCI-CTC grade; RR 1.83, 95% CI 1.12 to 2.99) [47]. In addition, Zhou's herbal formula combined with nicotinamide and vitamins B1 and B6 showed a significant improvement in the effectiveness rate in comparison to nicotinamide and vitamins B1 and B6 (researcher's own criteria; RR 1.53; 95% CI 1.14 to 2.06) [44]. In comparison, Ke's herbal formula [48] and the fumigation of a modified zhixiao xuanbi formula combined with a modified zhixiao xuanbi decoction did not show a significant improvement in the effectiveness rate [42]. However, Ke's herbal formula and the fumigation of a modified zhixiao xuanbi formula combined with a modified zhixiao xuanbi decoction were found to improve the sensory and motor nerve velocity significantly [42, 48]. Two trials reported QoL using the Chinese Anti-Cancer Association form, and the results of the intervention groups showed significant improvements in comparison to the control groups [40, 46].

### 3.9. Therapeutic Effects: Herbal Medicine for Oral Dosage Form

Here, two trials were investigated. One compared the bawei yangmai decoction and vitamin B12 [41]. The other compared goshajinkigan granules combined with vitamin B12 and with vitamin B12 alone [43]. The bawei yangmai decoction was found to improve the effectiveness rate in comparison to vitamin B12 (WHO grade; RR 1.77, 95% CI 1.12 to 2.79). However, the goshajinkigan granules combined with vitamin B12 did not show a statistical difference between the two groups. Regarding QoL, the bawei yangmai decoction was found to improve the QoL, as assessed by the CACA form, in comparison with vitamin B12 (MD 6.84, 95% CI 3.72 to 9.96). The goshajinkigan granules combined with vitamin B12 did not show a significant improvement on FACT-Taxane, in comparison to vitamin B12 alone. NCS was not reported.

### 3.10. Adverse Events

As shown in Tables 1 and 2, ten trials reported adverse events of interventions [30, 33, 36, 37, 39–41, 45–47], while the remaining 18 trials did not mention adverse events. One trial reported that two cases of adverse events occurred in the wenjing tongluo formula group [45] and the other nine trials reported no adverse events in the treatment and control groups. Eight trials reported adverse events associated with chemotherapy [21, 23, 24, 26, 28, 32, 35, 36] and seven reported no significant difference between the intervention groups and the control groups. One trial reported that Astragali Radix for intravenous infusion significantly improved chemotherapy induced anaemia in comparison to no treatment [26].

## 4. Discussion

We conducted this systematic review to assess the effectiveness of herbal medicine for CIPN in cancer patients. We searched 819 research studies through 13 English, Chinese, and Korean databases and ultimately identified 28 trials for inclusion with a total of 2,174 patients. Eighteen of the included trials investigated the preventive effects of herbal medicines (used either individually or in combination with conventional therapies), and ten trials tested the therapeutic effects of herbal medicines.

Five main outcomes were analysed to assess the effectiveness of herbal medicines combined with or in the absence of conventional therapies in relation to different comparators. Seventeen trials reported an incidence rate of CIPN assessed by various tools and 14 showed a significant difference between the two groups in the decrease of the incidence rate. Regarding clinical improvement, 12 trials reported it using various tools and 10 showed a significant difference between the two groups. Seven herbal formulas were associated with significant improvement in the rate of effectiveness, in comparison with the control groups. The NCS was studied in four trials, all of which indicated that different herbal medicines combined with/without conventional therapies had the advantage in terms of improving nerve conduction velocity. Nine trials reported QoL using KPS, the CACA form, or FACT-Taxane, and eight trials showed treatment groups superior to control groups in improving QoL. Ten trials reported adverse events (with two cases of adverse events occurring in one trial), and nine trials reported no adverse events. Nonetheless, we cannot conclude that the use of herbal medicines in CIPN is safe purely on the grounds that the remaining 18 trials did not report adverse events; in order for us to draw conclusions on safety, the documentation of adverse events must be in full compliance. In addition, eight trials reported adverse events associated with chemotherapy and seven reported no significant difference between the intervention groups and the control groups.

We found some noteworthy features in this review. A relatively large number of studies investigated and tested the external dosage form of herbal medicines, and approximately 15 different herbal formulas were investigated using hand and foot baths or fumigation. We found that due to the characteristics of CIPN—which is a kind of damage to the peripheral nervous system—the direct application of directly administering the herbal formula on lesions would be considered an effective dosage form. A significant variety of herbal formulas was investigated, with a total of 26 different formulas investigated in 28 trials. Noticeably, prescribing diverse herbal formulas for the same disease and modifying the herbs in a particular formula were very common in TEAM. Although these features would be useful in the clinical field, they additionally make it difficult to derive a conclusion regarding the effectiveness of the herbal medicines.

From this review, we found that herbal medicines, in combination with and without other therapies, have potentially preventive and/or therapeutic effects for CIPN. However, some limitations in the review prevent us from drawing clear conclusions. Although we planned to perform a meta-analysis in order to draw global conclusions about herbal medicine, the clinical heterogeneity induced by the different herbal formulas, different outcomes, and different comparators left us unable to pool the data. The participant sizes were also too small to draw conclusions about each herbal formula. A further issue is that, except for a few studies, such as Kono et al.'s research, the methodological quality of the studies was low; most of the included trials did not report sufficient information for random sequences and allocation concealment, and only four of them were performed in a double-blinded manner. As a result, biases might have occurred and influenced the apparent findings on the effectiveness of the herbal medicines.

In conclusion, this review found that herbal medicines showed potentially beneficial effects on CIPN. However, the small participant sizes and low methodological quality make it difficult to draw firm conclusions. More high-quality trials reporting sufficient methodological data should be conducted to corroborate findings of the studies in this review. In addition, more clinical homogeneous trials and further evidence of safety are required before drawing definitive conclusions concerning the effectiveness of herbal medicines.

## Figures and Tables

**Table 1:** Summary of the included randomised controlled trials of herbal medicines (preventive effect) for CIPN.

First author (year)	Sample size (dropouts): type of cancer	Treatment method			Main outcomes	Main results: (A) versus (B) (at end of treatment)	Effects estimate RR or MD (95% CI)
		Common treatment in both groups (regimen)	(A) Intervention group (regimen, participants)	(B) Control group (regimen, participants)			
*Herbal medicine for hand and foot baths or fumigation*							
Chen (2012)	120 (0): rectal cancer	FOLFOX (1 wk/cycle, ≥2 cycles)	Hand and foot baths of huoxue tongluo formula 20 min bid for 2 wk, *n* = 60)	Vitamin B12 (2 wk, *n* = 60)	(1) Incidence rate (NCI-CTC; sensory neuropathy)	(1) Grade 1/2/3/4 14/4/3/0 versus 36/7/3/0 (*p* < 0.01)	(1) Incidence rate (≥grade 1) 0.46 (0.31, 0.66)
Jiang (2013)	70 (0): gastrointestinal cancer	FOLFOX (3 wk/cycle for 6 cycles)	Hand and foot baths of herbal formula (bid for 2 wk, *n* = 34)	No additional Tx. (*n* = 36)	(1) Clinical improvement (researcher's own criteria)	(1) CR/PR/NP 26/6/2 versus 0/3/33 (*p* < 0.05)	(1) Effective rate 11.29 (3.81, 33.48)
Jiang (2015)	150 (0): colorectal cancer	Oxaliplatin based CTx. (3 wk/cycle, ≥2 cycles)	Hand and foot baths of siteng yixian formula (40 min bid during CTx., *n* = 75)	No additional Tx. (*n* = 75)	(1) Incidence rate (Levi's grade)	(1) Grade 0/1/2/3/4 (1 month after the end of treatment) 61/4/8/2/0 versus 33/7/22/13/0 (*p* < 0.05)	(1) Incidence rate (≥grade 1) 0.33 (0.20, 0.56)
Cai (2016)	50 (0): colorectal cancer	XELOX (3 wk/cycle for 2 cycles)	Hand and foot baths of wenjing huoxue formula (30 min qd during CTx., *n* = 25)	No additional Tx. (*n* = 25)	(1) Incidence rate (NCI-CTC; sensory neuropathy) (2) Adverse events (3) CTx. related A/E	(1) Grade 0/1~2//3~4 19/5/1 versus 10/11/4 (*p* < 0.05) (2) No A/E (3) NS	(1) Incidence rate (≥grade 1) 0.40 (0.19, 0.86)
Guo (2015)	71 (6): gastrointestinal cancer	Oxaliplatin based CTx.	Fumigation of herbal formula (20 min qd for 45 days, *n* = 32)	Warm water for hand and foot baths (20 min qd for *n* = 33)	(1) Incidence rate (NCI-CTC; sensory neuropathy) (2) QoL (KPS) (3) NCS (4) Adverse events	(1) Grade 0/1/2/3/4 0/16/14/2/0 versus 0/9/10/14/0 (*p* < 0.05) (2) 73.13 ± 4.98 versus 69.70 ± 5.68 (*p* < 0.05) (3) Significantly effective in NCS (4) No A/E	(1) Incidence rate (≥grade 3) 0.15 (0.04, 0.60) (2) 3.43 (0.84, 6.02)
Pan (2012)	48 (0): ovarian cancer	PC	(1) Chaihu jia longgu muli decoction (oral administration, 100 ml tid for 4 wk) (2) Hand and foot baths of yiqi huayu formula (20 min bid for 4 wk, *n* = 26)	No additional Tx. (*n* = 22)	(1) Incidence rate (WHO grade)	(1) Grade 1/2/3/4 6/4/0/0 versus 8/6/1/0 (*p* < 0.05)	(1) Incidence rate (≥grade 1) 0.56 (0.32, 0.99)
Li (2013)	126 (0): various types of cancer	(1) Oxaliplatin based CTx. (2) Vitamin B12 (0.5 mg tid for 7 days)	(1) Buyang huanwu decoction (bid for 30 days) (2) Hand and foot baths of herbal formula (20 to 30 min qd 30 days *n* = 66)	No additional Tx. (*n* = 60)	(1) Incidence rate (researcher's own criteria)	(1) Grade 1/2/3 54/9/3 versus 27/24/9 (*p* < 0.01)	(1) Incidence rate (≥grade 2) 0.33 (0.19, 0.58)

*Herbal medicine for intravenous infusion*							
Luo (2012)	90 (0): colorectal cancer	FOLFOX (4 wk/cycle for 6 cycles)	Astragali Radix for intravenous infusion (40 ml qd during CTx., *n* = 30)	(B1) (*n* = 30) No additional Tx. (B2) (*n* = 30) Glutathione IV infusion (1.8 g qd during CTx.)	(1) Incidence rate (Levi's grade) (2) QoL (KPS) (3) CTx. related A/E	(1) Grade 1/2/3/4 15/7/0/0 versus 10/12/3/4 versus 13/11/3/0 (2) 85.83 ± 6.85 versus 78.15 ± 6.56 versus 84.13 ± 6.41 (3) Significant improvement for anaemia in (A) compared with (B)	(1) Incidence rate (≥grade 1) (A) versus (B1): 0.74 (0.59, 0.92) (A) versus (B2): 0.81 (0.64, 1.04) (2) (A) versus (B1): 7.68 (4.29, 11.07) (A) versus (B2): 1.70 (−1.66, 5.06)

*Herbal medicine for oral dosage form*							
Nishioka (2011)	45 (0): advanced colon cancer	mFOLFOX 6 (2 wk/cycle for 20 cycles)	Goshajinkigan granules (7.5 g/day during CTx., *n* = 22)	No additional Tx. (*n* = 23)	(1) Incidence rate (DEB-NTC scale) (2) CTx. related A/E	(1) ≥Grade 3 (after 20 cycles) 33% versus 75% (*p* < 0.01) (2) NS	(1) No sufficient data
Abe (2013)	60 (0): breast cancer	Docetaxel based CTx.	Goshajinkigan granules (7.5 g/day during CTx., *n* = 33)	Vitamin B12 (1500 *μ*g/day, *n* = 27)	(1) Incidence rate (DEB-NTC scale) (2) Incidence rate (NCI-CTC; sensory neuropathy) (3) Pain (VAS) (4) CTx. related A/E	(1) Grade 1/2/3 2/5/6 versus 1/12/12 (*p* < 0.01) (2) Grade 1/2/3 7/6/0 versus 11/12/1 (*p* < 0.01) (3) 2.7 ± 2.2 versus 4.9 ± 2.4 (*p* < 0.01) (4) NS	(1) Incidence rate (≥grade 1) 0.43 (0.27, 0.66) (2) Incidence rate (≥grade 1) 0.44 (0.28, 0.69) (3) −2.20 (−3.38, −1.02)
Kono (2013)	93 (4): colon cancer	mFOLFOX6 (2 wk/cycle)	Goshajinkigan granules (7.5 g/day for 26 wk, *n* = 44)	No additional Tx. (*n* = 41)	(1) Incidence rate (NCI-CTC; sensory neuropathy) (2) Clinical symptom (FACT/GOG-Ntx-12) (3) Adverse events	(1) Grade 2/3 14/3 versus 17/6 (2) 7.0 versus 10.5 (*p* > 0.05) (3) No A/E	(1) Incidence rate (≥grade 3) 0.47 (0.12, 1.74)
Oki (2015)	186 (4): colon cancer	mFOLFOX6 (2 wk/cycle for 12 cycles)	Goshajinkigan granules (7.5 g/day during CTx., *n* = 89)	Placebo (*n* = 93)	(1) Incidence rate (NCI-CTC; sensory neuropathy) (2) CTx. related A/E	(1) Grade 0/1/2/3 5/39/30/15 versus 6/58/19/10 (2) NS	(1) Incidence rate (≥grade 1) 1.01 (0.94, 1.09)
Zhu (2007)	120 (0): gastrointestinal cancer	FOLFOX (2 wk/cycle for 6 cycles)	Guilong tongluo decoction (during CTx., *n* = 69)	No additional Tx. (*n* = 51)	(1) Incidence rate (Levi's grade) (2) QoL (KPS) (3) CTx. related A/E	(1) ≥Grade 1 17 versus 27 (*p* < 0.05) (2) Improvement of KPS scale 43/69 versus 25/51 (*p* < 0.05) (3) NS	(1) Incidence rate (≥grade 1) 0.47 (0.29, 0.76)
Liu (2013)	120 (0): colorectal cancer	FOLFOX4 (2 wk/cycle for 6 cycles)	Guilong tongluo decoction (bid during CTx, *n* = 60)	Placebo (*n* = 60)	(1) Incidence rate (NCI-CTC; sensory neuropathy) (2) Adverse events	(1) Grade 0/1 to 2/3 to 4 29/24/7 versus 18/23/19 (*p* < 0.05) (2) NS	(1) Incidence rate (≥grade 1) 0.74 (0.55, 0.99)
Sun (2008)	42 (0): gastric and rectal cancer	(1) FOLFOX (3 cycles) (2) Neurotropin (4 mg injection qd)	Yiqi huoxue decoction (4 wk, *n* = 20)	No additional Tx. (*n* = 22)	(1) Incidence rate (Levi's grade)	(1) Grade 1/2/3/4 2/1/0/0 versus 9/3/0/0 (*p* < 0.05)	(1) Incidence rate (≥grade 1) 0.25 (0.08, 0.75)
He (2010)	63 (1): gastrointestinal cancer	FOLFOX (3 wk/cycle for 6 cycles)	Yiqi wenjing tongluo decoction (during CTx., *n* = 32)	Glutathione IV infusion (1.8 g qd during CTx., *n* = 30)	(1) Incidence rate (Levi's grade) (2) QoL (KPS) (3) CTx. related A/E	(1) Grade 1/2/3/4 6/2/0/0 versus 10/5/1/0 (*p* < 0.05) (2) 80.38 ± 0.12 versus 67.16 ± 9.03 (*p* < 0.05) (3) NS	(1) Incidence rate (≥grade 1) 0.47 (0.24, 0.93) (2) 13.22 (9.99, 16.45)
Jia (2016)	80 (0): colorectal cancer	FOLFOX (2 wk/cycle for 8 cycles)	Shaoyao gouteng muer decoction (200 ml bid for 8 wk, *n* = 40)	No additional Tx. (*n* = 40)	(1) Incidence rate (NCI-CTC; sensory neuropathy) (2) QoL (KPS) (3) Adverse events	(1) Grade 0/1/2/3/4 (sum of 6 and 8 wk) 13/22/22/18/5 versus 4/13/14/35/14 (*p* < 0.01) (2) 80.19 ± 7.87 versus 77.47 ± 8.23 (*p* < 0.05) (3) No A/E	(1) Incidence rate (≥grade 1) 0.88 (0.79, 0.98) (2) 2.72 (−0.81, 6.25)
Xu (2016)	36 (0): gastrointestinal cancer	FOLFOX	Tanshinone II (80 mg qd during CTx., *n* = 18)	No additional Tx. (*n* = 18)	(1) Incidence rate (Levi's grade) (2) NCS (1) MNCV (2) SNCV	(1) Grade 0/1/2/3 13/4/1/0 versus 8/5/3/2 (*p* < 0.05) (2) 34.1 ± 3.5 versus 42.4 ± 3.9 (*p* < 0.05) (3) 38.5 ± 1.9 versus 43.5 ± 2.6 (*p* < 0.05)	(1) Incidence rate (≥grade 1) 0.50 (0.21, 1.17) (2) −8.30 (−10.72, −5.88) (3) −5.00 (−6.49, −3.51)

wk: week, FOLFOX: folinic acid, fluorouracil and oxaliplatin, bid: twice a day, NCI-CTC: National Cancer Institute Common Toxicity Criteria for Adverse Events, Tx.: treatment, CR: complete remission, PR: partial remission, NP: not perceptible, A/E: adverse events, NS: no significant differences between intervention and control groups, CTx.: chemotherapy, qd: once daily, XELOX: capecitabine and oxaliplatin, PC: paclitaxel and carboplatin, tid: three times a day, KPS: Karnofsky Performance Scale, IV: intravenous, DEB-NTC Scale: Neurotoxicity Criteria of Debiopharm, VAS: Visual Analogue Scale, FACT/GOG-Ntx: Functional Assessment of Cancer Therapy/Gynecological Oncology Group-Neurotoxicity, NCS: nerve conduction study, MNCV: motor nerve conduction velocity, SNCV: sensory nerve conduction velocity, and QoL: quality of life.

**Table 2:** Summary of the included randomised controlled trials of herbal medicines (therapeutic effect) for CIPN.

First author (year)	Sample size (dropouts): type of cancer; type of CTx.	Treatment method			Main outcomes	Main results: (A) versus (B) (at end of treatment)	Effects estimate RR or MD (95% CI)
		Common treatment in both groups	(A) Intervention group (regimen, participants)	(B) Control group (regimen, participants)			
*Herbal medicine for hand and foot baths or fumigation*							
Jia, 2009	60 (2): gastrointestinal cancer, FOLFOX4	n.c.	Hand and foot baths of wenloutong formula (30 min bid for 7 days, *n* = 30)	Warm water for hand and foot baths (30 min bid for 7 days, *n* = 28)	(1) Clinical improvement (NCI-CTC; sensory neuropathy) (2) Adverse events	(1) CR/PR/NP 25/3/2 versus 6/3/19 (*p* < 0.01) (2) No adverse events	(1) Effective rate 2.90 (1.68, 5.02)
Han (2011)	63 (2): various types of cancer, various types of regimen	n.c.	Hand and foot baths of wenjing huoxue tongluo formula (30 min qd for 2 wk, *n* = 31)	Warm water for hand and foot baths (30 min qd for 2 wk, *n* = 30)	(1) Clinical improvement (NCI-CTC; sensory neuropathy) (2) QoL (CACA form) (3) Adverse events	(1) PR/NP 26/5 versus 13/17 (*p* < 0.01) (2) 42.03 ± 4.52 versus 38.90 ± 5.65 (*p* < 0.05) (3) No adverse events	(1) Effective rate 1.94 (1.25, 3.00) (2) 3.13 (0.56, 5.70)
Lou (2014)	102 (1): various types of cancer, oxaliplatin	n.c.	Hand and foot baths of wenjing tongluo formula (20 min, bid for 7 days, *n* = 67)	Hand and foot baths of placebo (20 min, bid for 7 days, *n* = 34)	(1) Clinical improvement (NCI-CTC; sensory neuropathy) (2) NRS (pain) (3) NCCN (pain) (4) Adverse events	(1) CR/PR/NP 24/27/17 versus 2/10/22 (*p* < 0.01) (2) 2.40 ± 2.40 to 4.35 ± 2.39 (*p* < 0.01) (3) 11.81 ± 13.66 to 25.44 ± 16.79 (*p* < 0.01) (4) 2 cases in intervention group	(1) Effective rate 2.13 (1.32, 3.42) (2) −1.95 (−2.94, −0.96) (3) −13.63 (−20.15, −7.11)
Wei (2014)	66 (2): various types of cancer, various types of CTx.	n.c.	Hand and foot baths of wenyang tongbi formula (30 min qd for 2 wk, *n* = 33)	Vitamin B12 (30 min qd for 2 weeks, *n* = 31)	(1) Clinical improvement (NCI-CTC; sensory neuropathy) (2) QoL (CACA form) (3) Adverse events	(1) PR/NP 29/4 versus 19/12 (*p* < 0.05) (2) Improvement/stabilisation/decent 8/18/7 versus 3/13/15 (*p* < 0.05) (3) No adverse events	(1) Effective rate 1.43 (1.05, 1.95) (2) Improvement rate 2.51 (0.73, 8.60)
Shen (2015)	60 (0): various types of cancer, oxaliplatin	Vitamin B12 (0.5 mg tid for 2 wk), aidi injections and thymalfasin (2 wk)	Hand and foot baths of modified huangqi guizhi wuwu formula (30 min qd for 2 wk, *n* = 30)	No additional Tx. (*n* = 30)	(1) Clinical improvement (NCI-CTC; sensory neuropathy) (2) Adverse events	(1) PR/NP 22/8 versus 12/18 (*p* < 0.05) (2) No adverse events	(1) Effective rate 1.83 (1.12, 2.99)
Ke (2016)	40 (0): multiple myeloma, n.r.	Vitamin B12 (0.5 mg, tid for 30 days)	Hand and foot baths of herbal formula (30 min bid for 30 days, *n* = 20)	No additional Tx. (*n* = 20)	(1) Clinical improvement (NCI-CTC; sensory neuropathy) (2) NCS (1) MNCV: median nerve (2) MNCV: fibular nerve (3) SNCV: median nerve (4) SNCV: fibular nerve	(1) PR/NP 17/3 versus 14/6 (*p* < 0.05) (2.1) 53.9 ± 2.2 versus 46.1 ± 1.3 (*p* < 0.05) (2.2) 49.9 ± 2.7 versus 40.2 ± 2.0 (*p* < 0.05) (2.3) 48.7 ± 1.3 versus 41.8 ± 2.7 (*p* < 0.05) (2.4) 44.1 ± 2.9 versus 36.1 ± 1.8 (*p* < 0.05)	(1) Effective rate 1.21 (0.86, 1.71) (2.1) 7.80 (6.68, 8.92) (2.2) 9.70 (8.23, 11.17) (3.1) 6.90 (5.59, 8.21) (3.2) 8.00 (6.50, 9.50)
Chen (2012)	48 (0): gastrointestinal cancer, various types of regimen	Lipoic acid and vitamin B12 (qd for 2 wk)	(1) Modified zhixiao xuanbi decoction (bid for 2 wk) (2) Fumigation of modified zhixiao xuanbi formula (2 wk, *n* = 23)	No additional Tx. (*n* = 25)	(1) Clinical improvement (researcher's own criteria) (2) NCS (1) MNCV: median nerve (2) MNCV: fibular nerve (3) SNCV: median nerve (4) SNCV: fibular nerve	(1) PR/NP 19/4 versus 15/10 (*p* < 0.05) (2.1) 47.9 ± 4.28 versus 42.3 ± 5.16 (*p* < 0.05) (2.2) 47.1 ± 5.11 versus 42.5 ± 5.09 (*p* < 0.05) (2.3) 40.4 ± 5.02 versus 35.7 ± 4.74 (*p* < 0.05) (2.4) 38.8 ± 5.16 versus 34.5 ± 5.19 (*p* < 0.05)	(1) Effective rate 1.38 (0.95, 2.00) (2.1) 5.60 (2.93, 8.27) (2.2) 4.60 (1.71, 7,49) (3.1) 4.70 (1.93, 7.47) (3.2) 4.30 (1.37, 7.23)
Zhou (2013)	74 (0): gastrointestinal cancer, oxaliplatin	Nicotinamide, vitamin B1 and B6 (20 days)	Fumigation of herbal formula (30 to 40 min qd for 20 days, *n* = 38)	No additional Tx. (*n* = 36)	(1) Clinical improvement (researcher's own criteria)	(1) Complete recovery/prominent effect/good effect/ineffectuality 12/15/7/4 versus 5/12/4/15 (*p* < 0.05)	(1) Effective rate 1.53 (1.14, 2.06)

*Herbal medicine for oral dosage form*							
Zhang (2011)	60 (0): various types of cancer, various types of regimen	n.c.	Bawei yangmai decoction (150 ml, bid for 8 wk, *n* = 30)	Vitamin B12 (0.5 mg tid for 8 wk, *n* = 30)	(1) Clinical improvement (WHO grade) (2) QoL (CACA form) (3) Adverse events	(1) Effective rate 23/30 versus 13/30 (*p* < 0.01) (2) 45.07 ± 6.29 versus 38.23 ± 6.04 (*p* < 0.01) (3) No adverse events	(1) Effective rate 1.77 (1.12, 2.79) (2) 6.84 (3.72, 9.96)
Kaku (2012)	31 (2): ovarian or endometrial cancer, PC	Vitamin B12 (1500 *μ*g/day for 6 wk)	Goshajinkigan granules (7.5 g/day for 6 wk, *n* = 15)	No additional Tx. (*n* = 14)	(1) Clinical improvement (NCI-CTC) (1) Motor neuropathy (2) Sensory neuropathy (2) QoL (FACT-Taxane) (3) VAS (pain) (4) CTP	(1.1) 0.8 ± 0.9 versus 0.8 ± 1.1 (*p* > 0.05) (1.2) 1.4 ± 0.5 versus 1.3 ± 1.0 (*p* > 0.05) (2) 8.2 ± 7.0 versus 8.9 ± 8.4 (*p* > 0.05) (3) 3.4 ± 2.7 versus 3.7 ± 3.4 (*p* > 0.05) (4) NS	(1.1) 0.00 (−0.73, 0.73) (1.2) 0.10 (−0.48, 0.68) (2) −0.70 (−6.35, 4.95) (3) −0.30 (−2.54, 1.94)

wk: week, FOLFOX: folinic acid, fluorouracil, and oxaliplatin, n.c.: no common treatment, bid: twice a day, NCI-CTC: National Cancer Institute Common Toxicity Criteria for Adverse Events, CR: complete remission, PR: partial remission, NP: not perceptible, qd: once daily, QoL: quality of life, CACA: China Anti-Cancer Association, NCCN: National Comprehensive Cancer Network Guidelines for Adult Cancer, Tx.: treatment, NCS: nerve conduction study, tid: three times a day, MNCV: motor nerve conduction velocity, SNCV: sensory nerve conduction velocity, NS: no significant differences between intervention and control groups, FACT-Taxane: Functional Assessment of Cancer Therapy-Taxane, VAS: Visual Analogue Scale, and CTP: current perception threshold.

**Table 3:** Composition of the herbal formulas for CIPN in this review.

First author, year	Composition (daily dosage)
Nishioka, 2011; Kaku, 2012; Abe, 2013; Koto, 2013; Oki, 2015	*Goshajinkigan granule*: Rehmanniae Radix (10.7%), Achyranthis Radix (6.4%), *Corni fructus* (6.4%), Moutan Cortex (6.4%), Alismatis Rhizome (6.4%), Dioscoreae Rhizome (6.4%), Plantaginis Semen (6.4%), Poria (6.4%), processed Aconiti Tuber (2.1%), Cinnamomi Cortex (2.1%) (a mixture of aqueous extracts in fixed proportions)

Zhu, 2007; Liu, 2013	*Guilong tongluo decoction*: Ramulus Cinnamomi (9), Earthworm (12), Radix Astragali (30), Safflower (10), Radix Angelicae sinensis (12), *Ligusticum* (12), *Spatholobus* (30), Radix Paeoniae alba (30), Rhizoma Curcumae (9), licorice (6)

Sun, 2008	*Yiqi Huoxue decoction*: Astragali Radix (30), Angelicae Gigantis Radix (15), Spatholobi Caulis (15), Persicae Semen (10), Paeoniae Radix Rubra (10), Carthami Flos (10), Ligustici Rhizoma (10), Pheretimae Corpus (10), Curcumae Longae Rhizoma (10) Addition for numbness: *Trachelospermi caulis* (15), Chaenomelis Fructus (10) Addition for numbness of upper limb: Mori Ramulus (15), Osterici Radix (15) Addition for numbness of lower limb: Angelicae Pubescentis Radix (15), Achyranthis Radix (15)
He, 2010	*Yiqi wenjing tonglou decoction*: Astragali Radix (30), Cinnamomi Ramulus (12), Angelicae Gigantis Radix (12), Radix Paeoniae (12), Spatholobi Caulis (10), Pheretimae Corpus (10), Ligustici Rhizoma, Poria (10), Zingiberis Rhizoma Recens (6)
Chen, 2012	*Huoxue tongluo formula*: Zanthoxyli Pericarpium (10), Lycopodii Herba (30), *Phryma leptostachya* L. var. *asiatica* (30), Artemisiae Argyi Folium (20), Carthami Flos (10), Paeoniae Radix Rubra (10), Zingiberis Rhizoma Siccus (10), Angelicae Gigantis Radix (20), Pheretimae Corpus (10), Cinnamomi Ramulus (10), Lonicerae Flos (10), Olibanum (20), Myrrha (20)
Luo, 2012	Astragali Radix 40 ml (equivalent capacity of 80 g)
Pan, 2012	*(1) Chaihu jia longgu muli decoction:* Pseudostellariae Radix (15), Pinelliae Rhizoma (10), Glycyrrhizae Radix (10), Scutellariae Radix (15), Bupleuri Radix (10), Fossilia Ossis Mastodi (30), Ostreae Concha (30), Oldenlandiae Diffusae Herba (20), Scutellaria Herba (20), Fritillariae Thunbergii Bulbus (20) *(2) Yiqi huayu formula*: Astragali Radix (30), Angelicae Gigantis Radix (15), Paeoniae Radix Rubra (15), Pheretimae Corpus (15), Ligustici Rhizoma (10), Carthami Flos (15)
Li, 2013	*(1) Buyang huanwu decoction*: Astragali Radix (15), Angelicae Gigantis Radix (15), Spatholobi Caulis (15), Persicae Semen (15), Paeoniae Radix Rubra (10), Carthami Flos (10), Ligustici Rhizoma (10), Pheretimae Corpus (10) *(2) Herbal formula for hand and foot baths*: Taraxaci Herba (30), *Semiaquilegia adoxoides* (20), Chrysanthemi Indici Flos (20), Forsythiae Fructus (20), Scrophulariae Radix (20), Cicadae Periostracum (15), Sophorae Radix (20), Rehmanniae Radix (15), Cnidii Fructus (20), Phellodendri Cortex (15), Coptidis Rhizoma (6), Atractylodis Rhizoma (15), Kochiae Fructus (20)
Guo, 2015	Astragali Radix (40), Geranium Herba (40), Cinnamomi Ramulus (40), Artemisiae Argyi Folium (40), Zedoariae Rhizoma (40), Carthami Flos (40), Ligustici Rhizoma (40), Cyathulae Radix (40), Achyranthis Radix (40), Paeoniae Radix Rubra (40)
Jiang, 2015	*Siteng yixian formula*: Piperis Futokandsurae Caulis (30), *Trachelospermi caulis* (30), Uncariae Ramulus et Uncus (30), Spatholobi Caulis (30)
Cai, 2016	*Wenjing Huoxue formula*: Cinnamomi Ramulus (30), Lycopodii Herba (30), Angelicae Gigantis Radix (30), Blumeae Herba (30), Menthae Herba (15), Biotae Cacumen (30), Liquidambaris Resina (30), Ligustici Rhizoma (10)
Jia, 2016	*Shaoyao gouteng muer decoction*: Paeoniae Radix Alba (30), Uncariae Ramulus et Uncus (30), Glycyrrhizae Radix (9), Pruni Humilis Semen (6), Lactucae Semen (10), Auriculariae Polyporus (15), Gastrodiae Rhizoma (6), Scorpio (6), Bombycis Corpus cum Batryticatus (9)
Xu, 2016	Tanshinone IIA
Jia, 2009	*Wenloutong formula*: Carthami Flos, Aconiti Tuber, etc. (dosage not available)
Han, 2011	*Wenjing huoxue tonglou formula*: Aconiti Tuber Praepareta (40), Cinnamomi Ramulus (40), Ligustici Rhizoma (40), Epimedi Herba (40), Phrymae Herba (40)
Zhang, 2011	*Baweiyangmai decoction*: Astragali Radix (60), Angelicae Gigantis Radix (25), Spatholobi Caulis (20), Cinnamomi Ramulus (15), Pheretimae Corpus (10), Visci Herba (10), Achyranthis Radix (10), Glycyrrhizae Radix (10)
Chen, 2012	*(1) Modified zhixiao xuanbi decoction*: Astragali Radix (60), Rehmanniae Radix (30), Angelicae Gigantis Radix (10), Ligustici Rhizoma (25), Paeoniae Radix (15), Cinnamomi Ramulus (6), Hirudo (6), Carthami Flos (15), Pheretimae Corpus (15), Achyranthis Radix (20), Glycyrrhizae Radix (3), Zingiberis Rhizoma Recens (3) *(2) Fumigation of modified zhixiao xuanbi formula*: Astragali Radix (60), Rehmanniae Radix (30), Angelicae Gigantis Radix (10), Ligustici Rhizoma (25), Paeoniae Radix (15), Cinnamomi Ramulus (6), Hirudo (6), Carthami Flos (15), Pheretimae Corpus (15), Achyranthis Radix (20), Glycyrrhizae Radix (3), Zingiberis Rhizoma Recens (3), Sinapis Semen (20), Zingiberis Rhizoma Siccus (20), Zanthoxyli Pericarpium (20)
Zhou, 2013	Angelicae Gigantis Radix (30), Mori Folium (20), Spatholobi Caulis (20), Carthami Flos (10), Lycopodii Herba (10), Achyranthis Radix (15), Lycii Fructus (20), Perilla Folium (20), Pini Lignum (20), *Homalomena occulta* (15), Clematidis Radix (15), Angelicae Pubescentis Radix (15), Cynanchi Paniculati Radix (15), Astragali Radix (20), Acanthopanacis Cortex (15), Photiniae Folium (15)
Wei, 2014	*Wenyang tongbi formula*: Angelicae Pubescentis Radix (10), Taxilli Ramulus (10), Gentianae Macrophyllae Radix (10), Angelicae Gigantis Radix (10), Paeoniae Radix Rubra (10), Asari Herba Cum Radix (3), Cinnamomi Cortex Spissus (6), Hirudo (3), Bombycis Corpus cum Batryticatus (10), Scorpio (3)
Lou, 2014	*Wenjing tongluo formula*: Geranium Herba, Aconiti Tuber, Cinnamomi Ramulus, Carthami Flos (the proportions are 4 : 2 : 3 : 2)
Shen, 2015	*Modified huangqi guizhi wuwu formula*: Astragali Radix (50), Paeoniae Radix Alba (15), Cinnamomi Ramulus (12), Zingiberis Rhizoma Siccus (10), Zizyphi Fructus (10), Angelicae Gigantis Radix (12), Carthami Flos (10), Spatholobi Caulis (30), Clematidis Radix (10)
Ke, 2016	Sappan Lignum (30), Cinnamomi Ramulus (12), Lycopodii Herba (30), Salviae Miltiorrhizae Radix (30), Angelicae Pubescentis Radix (30), Osterici Radix (30)

## Appendix

### A. MEDLINE (via Ovid) Search Strategy

  #1. Exp Antineoplastic Agents/
  #2. (chemotherapy or (antineoplastic agents) or (chemotherapeutic Anticancer drug^*∗*^) or (anticancer agent^*∗*^) or cisplatin or carboplatin or oxaliplatin or bortezomib or docetaxel or paclitaxel or taxane or taxotere or (organoplatinum compounds) or platinum or vincristine or (vinca alkaloids) or thalidomide or (proteasome inhibitor)).mp.
  #3. #1 or #2
  #4. Exp Peripheral Nervous System Disease/
  #5. (neuralgia or paresthesia or hyperalgesia or (chemotherapy induced peripheral neuropathy) or CIPN or (peripheral neuropathy) or Polyneuropath^*∗*^ or (chemotherapy induced neurotoxicity)).mp.
  #6. #4 or #5
  #7. exp Drugs, Chinese herbal/
  #8. exp Herbal Medicine/
  #9. exp Plants, Medicinal/
  #10. exp Medicine, Chinese Traditional/
  #11. (traditional Korean medicine or traditional Chinese medicine or Traditional oriental medicine or Kampo medicine or alternative medicine or complementary medicine or herb^*∗*^ or herbal^*∗*^ or decoction^*∗*^ or botanic^*∗*^).mp.
  #12. or/#7–#11
  #13. exp Randomized Controlled Trials as Topic/
  #14. exp controlled clinical trials as topic/
  #15. (randomized controlled trial^*∗*^ or controlled clinical trial^*∗*^ or randomized^*∗*^ or randomly^*∗*^ or placebo or clinical trial^*∗*^ or controlled trial^*∗*^).mp.
  #16. or/#13–#15
  #17. #3 and #6 and #12 and #16.

### B. CNKI Search Strategy (in Chinese)

Consider the following: (chemotherapy) and (peripheral neuropathy or secondary neuropathy) and (traditional Chinese medicine or integrative medicine or Oriental medicine or Chinese herbal medicine or herbal medicine or decoction or pill or granule or injection or oral liquid or Chinese traditional patent medicine or drink) and (random or control).
